# Molecular patterns of sex determination in the animal kingdom: a comparative study of the biology of reproduction

**DOI:** 10.1186/1477-7827-4-59

**Published:** 2006-11-13

**Authors:** Panagiota Manolakou, Giagkos Lavranos, Roxani Angelopoulou

**Affiliations:** 1 Experimental Embryology Unit, Department of Histology and Embryology, Medical School, Athens University, Greece

## Abstract

Determining sexual fate is an integral part of reproduction, used as a means to enrich the genome. A variety of such regulatory mechanisms have been described so far and some of the more extensively studied ones are being discussed.

For the insect order of Hymenoptera, the choice lies between uniparental haploid males and biparental diploid females, originating from unfertilized and fertilized eggs accordingly. This mechanism is also known as single-locus complementary sex determination (slCSD). On the other hand, for Dipterans and Drosophila melanogaster, sex is determined by the ratio of X chromosomes to autosomes and the sex switching gene, sxl. Another model organism whose sex depends on the X:A ratio, Caenorhabditis elegans, has furthermore to provide for the brief period of spermatogenesis in hermaphrodites (XX) without the benefit of the "male" genes of the sex determination pathway.

Many reptiles have no discernible sex determining genes. Their sexual fate is determined by the temperature of the environment during the thermosensitive period (TSP) of incubation, which regulates aromatase activity. Variable patterns of sex determination apply in fish and amphibians. In birds, while sex chromosomes do exist, females are the heterogametic (ZW) and males the homogametic sex (ZZ). However, we have yet to decipher which of the two (Z or W) is responsible for the choice between males and females.

In mammals, sex determination is based on the presence of two identical (XX) or distinct (XY) gonosomes. This is believed to be the result of a lengthy evolutionary process, emerging from a common ancestral autosomal pair. Indeed, X and Y present different levels of homology in various mammals, supporting the argument of a gradual structural differentiation starting around the SRY region. The latter initiates a gene cascade that results in the formation of a male. Regulation of sex steroid production is also a major result of these genetic interactions. Similar observations have been described not only in mammals, but also in other vertebrates, emphasizing the need for further study of both normal hormonal regulators of sexual phenotype and patterns of epigenetic/environmental disruption.

## Background

Sex determination is an integral part of reproduction and an essential process for the evolvement and enrichment of the genome. It has thus been the subject of many studies in reference to species across the entire animal kingdom. From insects to mammals, there is much to learn from the many mechanisms employed to determine sexual fate. This is no lost cause, since the study of sex determination and differentiation is only the natural expansion of comparative biology and reproductive physiology in the modern, molecular Era. Interestingly, data so far accumulated by a variety of model organisms has shown a relative economy in the molecular regulation of sex determination. More specifically, sex determination has so far proven to be a result of one of the following three mechanisms:

a) Environmental action on the embryo at a crucial stage of development. To the extent that this interaction is associated with temperature alterations, the process is also described as temperature-dependent sex determination and the developmental stage of sex determination is referred to as the thermosensitive period (TSP). This mechanism is mainly observed in reptiles and fish.

b) Genetic action, when at least one specific gene is considered to be the central regulator in a cascade of events leading to the determination of sexual phenotype. This mechanism is already known to apply in the case of several animals, including invertebrates (insects, worms) and amphibians. Moreover, it is a proposed regulatory mechanism for several species, whose study has so far been limited or led to inconclusive data as to the attempt to detect a single, specific, sex-determining gene.

c) The presence of distinct sex chromosomes or gonosomes. The identical pair may be present in both males (birds) and females (mammals) and their major sex-determining gene may be either known (e.g. mammalian SRY) or still suspected [1-3].

Although sex determination has been suggested to promote specific functions at a universal level, such as selective cell proliferation (Mittwoch) or steroid hormone accumulation (Howard), this issue remains debatable [1,4]. What is even more intriguing is the fact that the conservation of relatively limited regulatory patterns in sex determination may suggest the presence of a single general regulatory scheme, at least in vertebrates, potentially involving or incorporating both hormonal elements and dosage compensation epigenetic regulatory phenomena, whenever necessary [5]. Such a discovery would bear great implications for comparative biology studies and might also allow important applications in the field of reproductive endocrinology. The study of more model organisms is a necessity to investigate this hypothesis and the consolidation of both recent and classic data from the relevant research work may significantly facilitate this discussion. This essay is dedicated to the brief and yet compact presentation of some of the better studied animal models of sex determination, in an attempt to approach that knowledge.

## Invertebrates

### Hymenoptera

**Figure 1:**
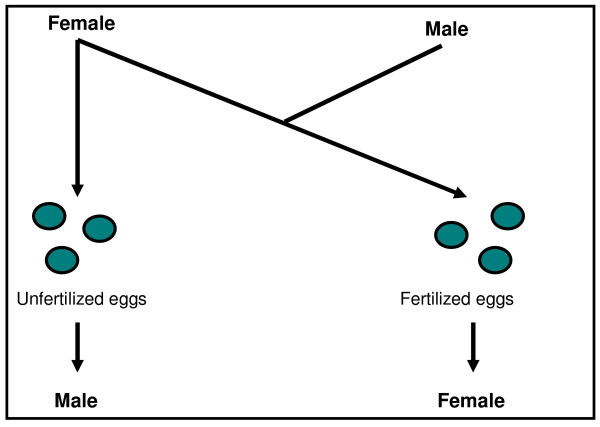
**Haplodiploid reproduction**. In Hymenoptera, unfertilized eggs develop into uniparental haploid males whereas fertilized eggs into biparental diploid females.

One such interesting mechanism is the haplodiploid genetic system we encounter in the insect order of Hymenoptera. More than 200,000 species of ants, bees and wasps are capable of laying both unfertilized eggs, that typically develop into uniparental (originating from one single female parent) haploid males, and fertilized eggs that can give us biparental (originating from two parents, male and female) diploid females (see Figure 1).

**Figure 2:**
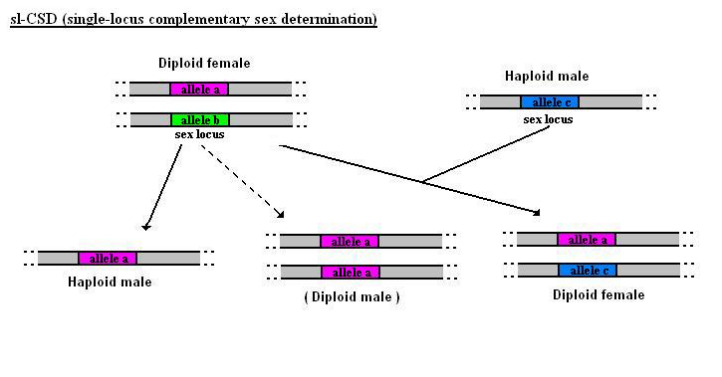
**Single-locus complementary sex determination (sl-CSD)**. In single-locus complementary sex determination (sl-CSD), heterozygotes at a single sex locus develop as females whereas hemizygotes and homozygous diploids develop as males. However, homozygous diploid males are generally sterile, unable to mate or not viable.

That can be accomplished with several strategies. One of the best understood seems to be single-locus complementary sex determination (sl-CSD), in which sex is determined by multiple alleles at a single locus. Heterozygotes at that sex locus develop as females whereas hemizygotes, and the odd case of homozygous diploids (i.e. through matched matings or faulty meiosis), develop as males (see Figure 2) thus providing us with the pattern presented above (see Figure 1).

In honeybees, for example, the sex locus has recently been identified as the *csd (complementary sex determiner) *gene that encodes an SR protein (Arginine-Serine rich protein) [6]. The initial observation that *csd *function was required only in females and that its product is nonfunctional when derived from only one allele [7] was followed by the suggestion of three possible models. First, that different allelic CSD proteins form active heterodimers. Second, that CSD proteins derived from the same allele form homomers, with two homomer species in females and one in males. And third, that merely the existence of different alleles is required in females for *csd *to complete its function [8].

**Figure 3:**
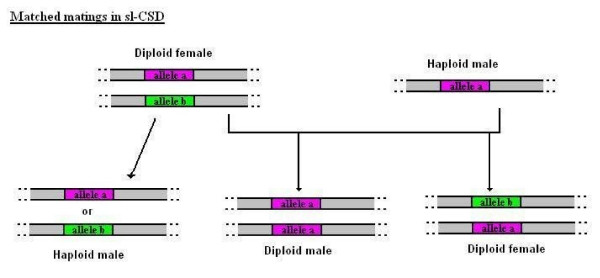
**Matched matings in sl-CSD**. In matched matings, half the diploid offspring are homozygous at the sex locus and turn into diploid males, which are unable to contribute to reproduction.

However, it should be noted that sl-CSD has been known to exhibit an evolutionary pressure against species with higher rates of inbreeding, due to one of its major faults. In most cases, mating leads to the creation of offspring with two different alleles at the sex locus (diploid females). However, a mating in such populations has higher probability of a union between a male and a female that share the same allele, a condition also known as a matched mating[9]. In matched matings half the diploid offspring are predicted to turn out homozygous at the sex locus and develop as males rather than females, whereas diploid males in species with sl-CSD are generally sterile, unable to mate or not viable (see Figure 3) [10]. Such is the example of the honeybee, where homozygous diploid males created from inbreeding are eaten by the workers [7].

### Dipterans (*Drosophila melanogaster*)

**Figure 4:**
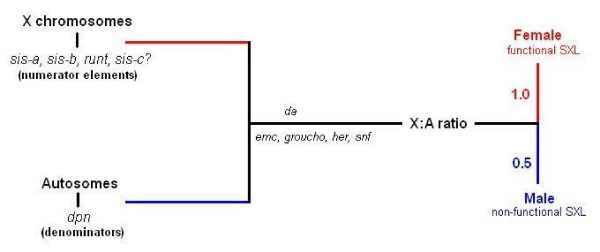
**The X:A ratio determines sex in *Drosophila melanogaster***. In *Drosophila melanogaster *sex is determined by the X:A ratio, which is communicated through the balance between the X numerator elements and the autosomal denominators in the presence of several maternally derived proteins. An X:A ratio of 0.5 leads to a non-functional SXL and male development, whereas an X:A ratio of 1 maintains SXL in its active state and is conducive to female development.

Taking things a step further, we enter the realm of Dipterans and *Drosophila melanogaster*, one of the model organisms in which the sex determination pathway has been elucidated in the greatest detail. Here the choice between male and female development is made by one single switch gene by the name of *sex-lethal *(*sxl*) in response to the ratio of X chromosomes to autosomes (X:A ratio) [11]. The latter is communicated early in development through the delicate balance between the dose-sensitive X chromosome numerator elements (those include genes such as *sis-a*, *sis-b*, *runt *and less so *sis-c*) and the autosomal denominators (such as *dpn*) in conjunction with the maternally derived products of the *da *gene and the more recently studied *emc*, *groucho, her *and *snf *(see Figure 4) [12]. We are not yet completely certain of the logistics of it, but it seems that the feminizing effect of the numerator elements is measured against the masculinising denominators, with the maternally derived products of the rest of the genes acting as point of reference.

**Figure 5:**
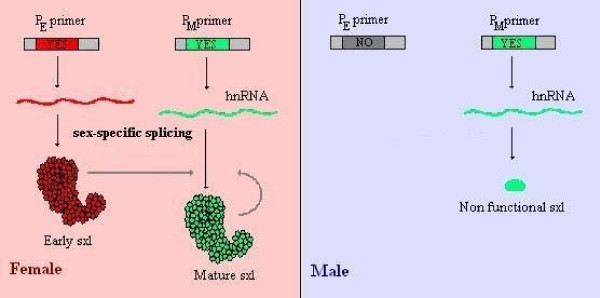
**Sex-specific splicing of the sxl mRNA**. Early activation of the *sxl *gene through a different primer (P_E_) in females allows the appearance of an early SXL protein that guides the splicing of the mRNA originating from the 'standard' primer (P_M_). This alternative splicing leads to a functional 'mature' SXL protein that then takes up the role of retaining its active state. In males, where no early transcripts can be found, a male-specific exon is included which contains many early stop codons thus leading to the creation of a truncated and non functional protein.

All this takes place early in development, leading to the activation of the *sxl *gene through an "early" promoter in females. This early form of the SXL protein, absent in males, then orchestrates a specific splicing of the mRNA produced through the activation of the "mature" promoter in females. In males, standard splicing of the *sxl *mRNA leads to a non-functional protein. It is only in females, through an autoregulatory feedback loop, that *sxl *manages to keep itself in an active state through this sex-specific splicing [11,13] (see Figure 5).

**Figure 6:**
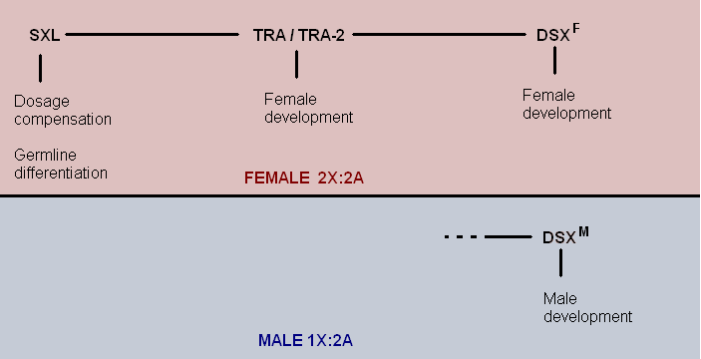
**Genes involved in sex determination in *Drosophila melanogaster***. The SXL protein regulates the female-specific splicing of the *tra *mRNA. The TRA protein then forms dimers with TRA-2 which regulate the sex-specific splicing of *dsx *mRNA. DSX^F ^is the result of said sex-specific splicing in females, whereas DSX^M ^is present in males. All of the above also interact with other genes in turn, in order to mediate sexual development.

Once the SXL active state has been established, it then goes on to regulate a series of other proteins that control female development, once again through the process of alternative splicing, leading finally to the two alternative products of the *doublesex *gene (*dsx*), DSX^F ^and DSX^M ^[14,15] (see Figure 6). The end-result? A series of intricate gene interactions that can take it from there and establish the development of the appropriate sex. Still, it is interesting to note, that the Y chromosome, present in males, takes no part in this entire process, and that its sole use is to help in the successful completion of the process of spermatogenesis later on in the differentiation of the male germline.

### Nematodes (*Caenorhabditis elegans*)

Another model organism that uses a single gene switch and the subsequent hierarchy of gene pathways to determine sex is the nematode *C.elegans*. Here again the animal's sexual fate depends on the X:A ratio, and there isn't even a Y chromosome present in males to later on interfere with the germline. However, *C.elegans *worms are special in that the choice lies between males with one X chromosome and hermaphrodites with two.

**Figure 7:**
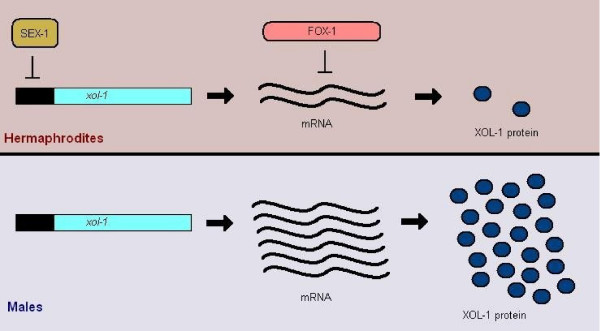
**The currently known X-signal elements in *C.elegans***. In *C.elegans*, the X-signal elements, such as the SEX-1 and FOX-1 proteins, control the levels of XOL-1 and help determine sex.

**Figure 8:**
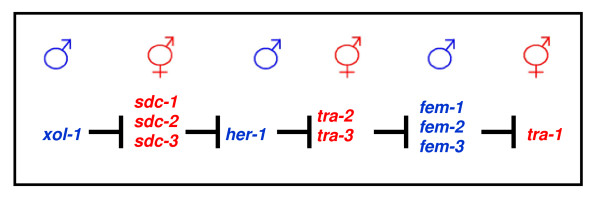
**Sex gene pathway in *C.elegans***. A simple depiction of the sex determination gene pathway as it is known today in the soma of *C.elegans*. The interactions between several groups of gene products that have been observed to have an inhibitory effect on each other follow the switch of *xol-1*. The result is that several of these proteins remain active only in males only and others only in hermaphrodites.

**Figure 9:**
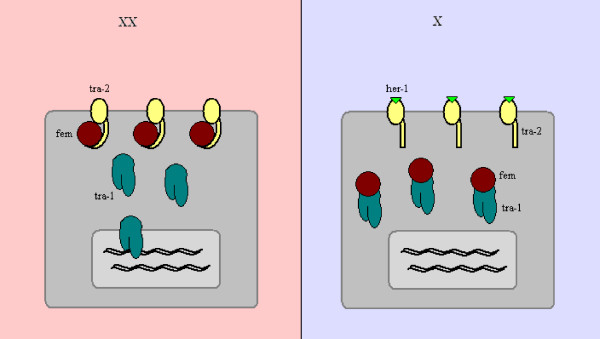
**Sex determining interactions on a cellular level**. Suggested protein interactions in the later stages of the sex determination pathway in *C.elegans*. While the SDC proteins have also been known to serve as part of the dosage compensation mechanism in *C.elegans*, HER-1 has been pictured as capable of binding to the TRA-2 receptor, which then releases the FEM molecules in males. Those in turn bind to the TRA-1 transcription factors rendering them inactive. In hermaphrodites, the TRA-2 receptors retain their hold on FEM, and TRA-1 is free to act as a transcription factor on the genome.

As before, the X:A ratio is communicated with the help of several "X-signal elements", such as the SEX-1 (signal element on X) protein that acts on the level of transcription and the FOX-1 (feminizing locus on X) protein that acts post-transcriptionally [16]. These two, among others that have yet to be deciphered, manage to suppress the levels of the XOL-1 (XO lethal) key protein, or what we could call the *C.elegans *sex switching gene [17] (see Figure 7). From there on, it is a matter of tracking down a pathway of inhibitory genes, to result at the TRA-1 (transformer) protein, free to act in hermaphrodites and regulate several other genes [18,19]. This pathway in fact involves several groups of gene products, some of which retain their active state in males and others in hermaphrodites (see Figure 8). One possible model incorporating these interactions is depicted in Figure 9, and includes the interaction between HER-1 and the TRA-2 receptor in males, which allows the FEM proteins to inhibit TRA-1 from acting as a transcription factor (see Figure 9).

**Figure 10:**
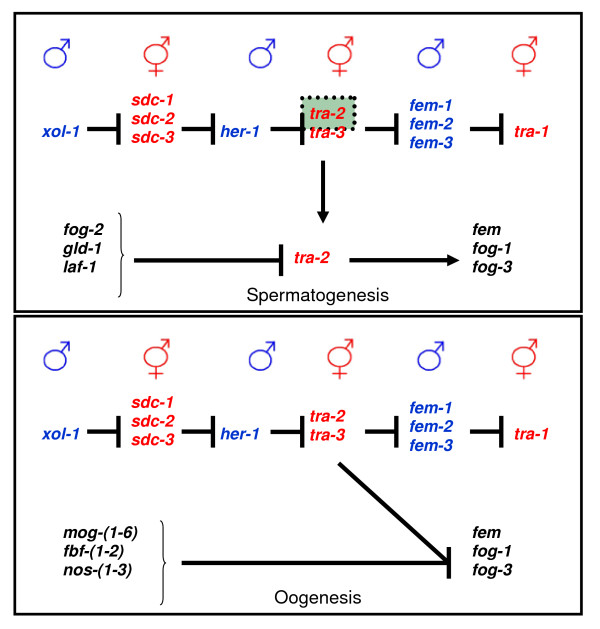
**Gene interactions that allow spermatogenesis and oogenesis in the hermaphrodite *C.elegans*, as opposed to the gene pathway in the soma**. The top half of each frame displays the gene pathway for sex determination in the *C.elegans *soma and the bottom half the changes that concern the hermaphrodite germline. During the fourth larval stage (L4), a special set of genes expressed in the germline (*fog-2, gld-1, laf-1*) allows spermatogenesis to occur in hermaphrodites by interfering with the original sex determination pathway (inhibition of *tra-2 *that leads to the activation of the *fem *gene products and others such as *fog-1 *and *fog-3*). Once spermatogenesis is over and the hermaphrodite enters its mature stage (M), the original sex determination pathway is re-established (*tra-2 *becomes active again) in the germline of adult hermaphrodites and makes the switch to oogenesis (by inactivating the genes *fem, fog-1 *and *fog-3 *gene products that allowed spermatogenesis).

However, the *C.elegans *hermaphrodites pose an interesting issue. These are specialized females which in the fourth and final larval stage (L4) produce around 300 sperm, to use for self-fertilization when there are no males available [20]. This requires a careful regulation of the switching between the male and female differentiation of the same germ cells without the benefit of the usual sex determination pathway, since the "male" genes that normally regulate spermatogenesis are inactive anyway. Instead, a new series of genes take over in a specific stage of development and act in place of the HER-1 protein to inhibit *tra-2 *and allow spermatogenesis to take place till the end of the L4 stage (see Figure 10). Once this is over and at the onset of adult life, a new series of genes take their place, *tra-2 *is once again active, and the adult hermaphrodite is free to continue with oogenesis for the rest of its life [19,21] (see Figure 10).

## Vertebrates

### Reptiles

The different species of reptiles present a considerable variety of sex determination patterns. For instance, most snakes possess a ZZ/ZW pattern of sex chromosomes, similar to that discussed later as the model mechanism for sex determination in birds. The study of lizards has led to more complex findings, with different species having either a ZZ/ZW sex chromosome pair or a XX/XY system, similar to that observed in mammals [22,23].

On the other hand, many species of reptiles, including most terrestrial turtles and all crocodilians and sea turtles examined to this date, have no discernible sex chromosomes, nor is their sex determined by the presence or absence of specific genes. In these organisms, it is the temperature of the environment in a specific period of incubation that can determine whether the animal in question will turn into a male or a female [24,25].

Indeed, studies have shown that there seem to be no significant differences in the expression of sex-related genes. Instead, there is a specific period of incubation, which is generally considered to lie in the middle third of development, during which the temperature of the eggs controls quite accurately their sexual fate. This particular period is also known as the thermosensitive period (TSP).

**Figure 11:**
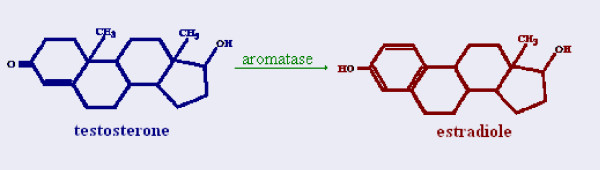
**Aromatase**. Aromatase is a cytP450 enzyme that allows the conversion of androgens into estrogens.

**Figure 12:**
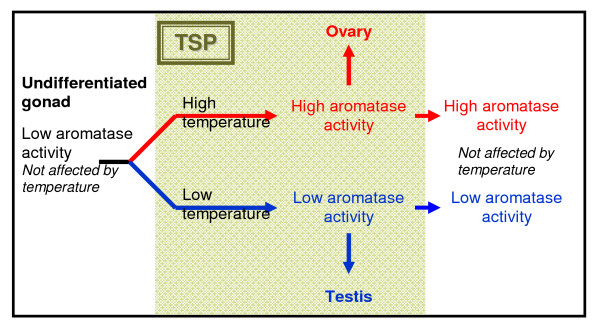
**Temperature-dependent sex determination**. Aromatase activity levels during the thermosensitive period (TSP) are regulated by the temperature of the environment and control gonadal differentiation. Changes in the environment temperature before and after TSP do not seem to affect sex.

It is during this period that a very specific enzyme enters into the equation. Aromatase, a cyt450 enzyme responsible for the conversion of androgens into estrogens is common among many organisms (see Figure 11). In reptiles, while steroidogenesis begins very early, prior even to the thermosensitive period, aromatase activity remains universally low. With the onset of the thermosensitive period however, aromatase activity seems to increase in certain temperatures, which vary for each species. For example, in marine and freshwater turtles, higher temperatures cause an exponential increase of aromatase activity, whereas in lower temperatures aromatase activity remains low. The different levels of aromatase activity then guide the differentiation of the indifferent gonad into an ovary or testis. Once the thermosensitive period is over and the fate of the gonad has been established, further changes in temperature seem to have no effects (see Figure 12) [26,27].

**Figure 13:**
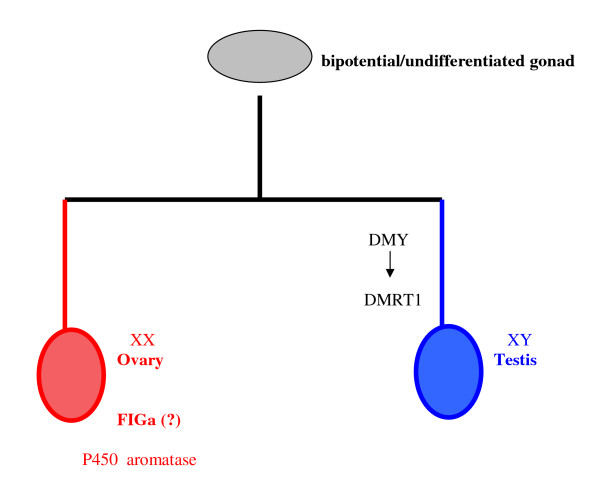
**Sex determination in the medaka**. Although many details for the molecular model of sex determination in the medaka are still missing, the mechanism is known to be based on the presence of XX/XY sex chromosomes. Following a stage of undifferentiated gonads, males exclusively express *DMY*, a gene bearing a DM domain, which is a genetic feature that considered central in sex determination pathways of various species. Among the genes induced downstream is *DMRT1*, which participates in gonadal development and differentiation in fish, birds and mammals. In XX females, the exact genetic cascade triggered in the absence of DMY is unclear, but it supposed to involve sex-specific gene expression, such as *FIGa *and sex steroid/aromatase regulation.

Interestingly, a number of genes originally described as part of the genetic regulation of sex development in men and other mammals have also been detected in reptiles. For instance, in the sea turtle *Lepidochelys olivacea*, several genes so far related to mammalian sex determination are expressed, including *DAX1 *(dosage-sensitive sex reversal 1), *DMRT1 *(*doublesex*- and *mab-3*-related transcription factor 1) and *SOX9 *(SRY related HMG box 9). In particular, *DAX1 *is a known regulator of gonadal development in mice and other mammals, considered to be an "anti-testis" gene, although this may approach may prove to be too simplified. In reptiles, the gene is not differentially expressed in response to temperature variation during the TSP, therefore, its role in reptile sex determination is unclear. The gene is also expressed in crocodilians with temperature-dependent sex determination, such as *Alligator mississippiensis*. Whether this gene could indeed be a target for androgen or estrogen-related actions following the TSP remains unknown. As far as *DMRT1 *is concerned, the gene was initially related to sex determination in *D. melanogaster*, due to the presence of a domain compatible to the sex determinant gene *DSX*. Subsequent research, however, has proven the gene's expression in several other species as well, including birds, fish and reptiles. In alligators, such as *A. mississippiensis*, the gene is expressed exclusively in the gonads of males. Moreover, its expression appears to precede that of *SOX9*, another testis-specific gene conserved in a vast number of species, ranging from reptiles to mammals. The latter gene is originally expressed in the bipotential gonad of reptile embryos, but following the TSP, it remains active only in males, making it a candidate gene for sex steroid-induced regulation. In alligators, *SOX9 *is also related to increased *AMH *(Anti-Müllerian Hormone) levels, but, contrary to mammals, *AMH *induction chronologically precedes that of *SOX9 *[3,23]. In the case of lizards, an attempt has also been made to examine sexual dimorphism in the brain. The first results from these experimental series show distinct differences in estrogen receptor expression and progesterone concentrations in specific areas of the central nervous system, a finding that may imply that aromatase regulation is only the first step in a sequence of several more complex sex-specific/dimorphic genetic phenomena that still remain to be examined [28].

Finally, it has recently been suggested that aromatase may also be regulated by secondary parameters, other than temperature. This has been described for instace, in the case of Prostaglandin E2, which appears to be associated with increased aromatase action [29]. Immunological reactions and cytokine levels may also be important. The latter has led to clinical applications in humans, with the attempt to treat oncological patients with hormone-sensitive cancer, with selective Interleukin-6 pharmaceutical modulators, thus indirectily aiming at aromatase suppression [30].

### Amphibians

The thermosensitivity of the gonads has been demonstrated not only in reptiles, but also in several fish and some amphibians. These tend to combine a genotypic sex determination mechanism -either male heterogamety, female heterogamety or polygenic- with the mechanism demonstrated above. The result is a phenomenon known as sex reversal, where the effects of temperature may go against the genotypic directions, allowing the existence of animals in genotypic and phenotypic sex discordance [27]. (Table 1)

**Table 1:** Sex reversal

	**Female-producing temperatures**	**Male-producing temperatures**
**XX (ZW)**	Female (in accordance with genotype)	Female OR male (in discordance with genotype)
**XY (ZZ)**	Male OR female (in discordance with genotype)	Female (in accordance with genotype)

The combination of genotypic and temperature-dependent sex determination allows a phenomenon known as sex reversal, where the phenotypic sex does not always agree with the genotypic directions.

In particular, male or female heterogamety has been described in various species of anurans and urodeles. Sex chromosomes of various types may be present, following both the XY/XX and WZ/ZZ pattern that usually apply to mammals and birds, respectively. The exact mechanism by which temperature regulates sex determination in amphibians is not yet deciphered, but it doesn't seem to apply to the TSP-aromatase regulation model of reptiles. Hormonal action may also act in the process of acquisition of sexual phenotype, either independently or in conjunction with temperature variation [3].

Gene studies in amphibian sex determination are not as extensive as in other animal models. Of the various genes so far associated with sex determination in other species, amphibians appear to express *DMRT1*. However, it is not yet clear whether this is a downstream product in the sex differentiation cascade or a factor with a more central role in sex determination [3,31,32].

### Fish

There are numerous species of fish in the animal kingdom, with estimations as to their current number reaching a mean price of 25.000. As one may easily perceive, among such a variety of living organisms, research has been focused on relatively few, specific model organisms, each of which has been considered representative of the reproductive physiology of several other closely related species. Among the mechanisms observed, one may refer to a) the presence of true hermaphrodites, a strategy usually associated with lower evolutionary levels (e.g. the previously described model of invertebrates-nematodes) b)temperature-dependent sex determination, with a process similar to the one known to be characteristic of most reptiles and c) sex chromosomes. The latter may follow either the XY/XX or the ZW/ZZ pattern [3,33].

Contrary to mammals, the sex determining genes have not yet been described in fish, although some candidacies have been proposed. It might also be possible that, instead of a common, uniform gene pattern for all fish, different genes will be proven to be the major sex determinants in every species. According to some researchers, it might also be possible to assume a number of competing genes in every species, with environmental and/or hormonal parameters regulating their relative priority in sex determination in every birth [33]. Of the various model organisms available for study, we will limit our reference to four characteristic examples, namely the atlantic salmon, the platyfish, the medaka and the zebra fish.

The atlantic salmon (*Salmo salar*) was, until recently, an organism within unknown genetic sex determinants. However, recent data has detected the candidate sex-determining locus of this species as part of chromosome 2. For this reason, this large metacentric chromosome is now regarded as the sex chromosome of this species. Research has now turned to the detailed study of the region, in an attempt to identify the exact position and structure of the single sex-determining gene, which has been proposed to exist within the aforementioned locus [34].

The platyfish (*Xiphophorus maculatus*)'s genome may contain any of three sex chromosomes, namely X, Y and W. This allows significantly more combinations in the population than those observed in other species, applying to the "traditional" principle of only two sex chromosome types available (ZW and XY pairs, respectively). Of all the combinations, WX, WY and XX develop as females, while XY and YY become males. No specific sex-determining gene has been described so far, although the W chromosome is considered a major candidate for its position, since its presence coincides with female phenotype regardless of the type of the second sex chromosome. However, some genes, previously described in other species and associated to reproductive physiology and development, are also found in this and other fish species. These include *SOX *family members, such as *SOX9 *and *DMRT1*. On the other hand, classical hormonal regulators of sex differentiation, such as AMH have not yet been identified in fish [3,33].

*DMRT1 *has been been shown to be particularly important for sex determination in the teleost medaka, *Oryzias latipes*. The sex determining system of the medaka is male heterogametic, i.e. it follows the XX/XY principle known from mammalian reproduction. Although some similarities with genes of the mammalian sex chromosomes may exist, the major sex determinant of mammals, i.e. *SRY *(sex determining region of the Y chromosome) is missing. Consequently, another, previously unknown, sex-determining gene must be present in the medaka genome. Indeed, in the Y chromosome of the fish a new gene has been detected, bearing six exons and a DM domain. The latter is a major characteristic of genes involved in sex determination in invertebrates, such as *doublesex *and *mab3 *in *D.melanogaster *and *C.elegans*, respectively. This new gene was named *DMY *(DM domain of the Y chromosome) and it is homologous to *DMRT1 *gene, which is conserved in various species. Although a lot of information is still missing, it appears that in the male, *DMY *and *DMRT1 *operate in procession as strong determinants of gonadal development. In the female, the role of aromatase is once again central, although its induction, in this case, may be a genetic rather than temperature-related event. Other genes' expression has also been detected exclusively in females, such as *FIGa *(factor in the germ line a), but their correlation with aromatase induction remains to be proven (see Figure 13) [35].

Finally, sex determination in the zebra fish is considered to be a genetic phenomenon, but the details of the process are still under examination. Of particular interest are recent data, proving the expression of two sex-related genes in the zebra fish [33,36,37]. These are a) *vasa*, a gene family expressed exclusively in the gonads of several species, including *D.melanogaster*, mice and fish and b) *FtzF1 *(fushi tarazu factor 1), a gene originally described in *Drosophila *and nkown to encode the steroidogenic factor 1 (*SF1*) in mammals, thus regulating sex steroid production [33,36].

### Birds

Next, approaching birds, we begin to tread on more familiar ground, as once again we return to sex chromosomes. In birds however, females are the heterogametic sex, carrying one copy of each of the so called Z and W sex chromosomes, whereas males are homogametic ZZ. The Z and W chromosomes have no relation to the mammalian X and Y, and in fact seem to have evolved from different pairs of autosomes. And this is part of the reason we are not yet certain which of the two carries the genetic trigger for sex determination [38,39].

To this day, there are two major theories under investigation. Sex may depend on Z chromosome dosage, according to the example of *Drosophila melanogaster *and *C.elegans*. One candidate gene for this theory is the *DMRT1*, which is located on Z chromosomes, escapes dosage compensation and is expressed specifically in the gonads, and is thus capable of linking the number of Z chromosomes with gonadal differentiation [40,41].

**Figure 14:**
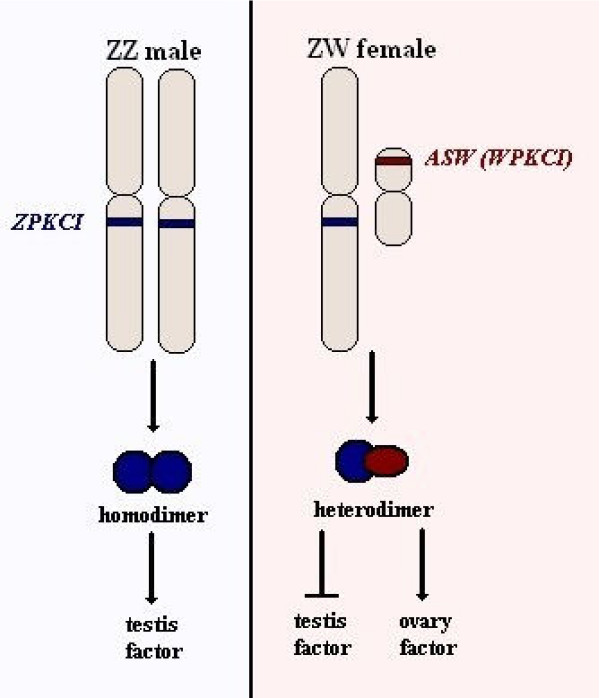
**The role of *ZPKCI *and *ASW (WPKCI) *in ZW sex determination**. According to one theory, the ZPKCI proteins form homodimers in ZZ males that stimulate a factor required for the differentiation of the testes. Whereas in ZW females, the ASW (also known as WPKCI) proteins form heterodimers with ZPKCI that may prevent the activation of that factor or stimulate directly the differentiation of ovaries.

On the other hand, sex may be determined by the feminizing presence of the W chromosome, following the example of Y in eutherian mammals. There are two different mechanisms that are being studied and can support this theory. One includes the *FET1 *gene, which is located on W, does not have a Z homologue and is expressed almost exclusively in the female urogenital system [18]. The other includes the *ASW *gene, also known as *WPKCI*, and its Z homologue *ZPKCI*, since it has been proposed that the products of those two genes are capable of dimerisation, with a ZPKCI homodimer acting as a testis factor and a WPKCI/ZPKCI heterodimer preventing this effect (see Figure 14) [39-41].

One way to discern between the two theories would be to look into different combinations of Z and W chromosomes. Indeed, scientists have studied ZW aneuploidy in an effort to better understand how things work. It turns out that ZZZ animals develop testes but are infertile, ZWW animals die early in embryonic development, but ZZW combinations manifest as intersexual: the animals appear female on hatching, but slowly turn into males at sexual maturity. It is still possible, thus, that a combination of the above is in fact applied [40,42].

## Mammals

### Marsupials

That final idea was borrowed by the mechanism applied in marsupials. Here we have a set of X and Y chromosomes related to those found in eutherian mammals. The basic marsupial Y chromosome is the smallest of any mammal but retains its ability to turn the undifferentiated gonads into testes [22]. However, the differentiation of the embryonic testis does not also control all aspects of sex differentiation. The formation of the mammary glands and scrotum develops before gonadal differentiation takes place and is independent of gonadal hormones [43]. In fact, it appears to be under the control of genes located on the X chromosome. So it happens that XXY animals have testes, but a pouch with mammary glands has replaced their scrotum, whereas XO animals have no testes, but an empty scrotum in place of a pouch (Table 2) [43,44]. These X-linked genes have yet to be identified, but already the autosomal *SOX9 *has been reported of being expressed in the scrotum and mammary primordial before birth [45].

**Table 2:** Different aspects of sex differentiation in marsupials

	**Y chromosome (testes)**	**No Y chromosome (absence of testes)**
**One X chromosome (scrotum)**	**XY**	**XO**
	Male animals with scrotum and testes	Animals without testes and an empty scrotum instead of a pouch
**Two X chromosomes (pouch and mammary glands)**	**XXY**	**XX**
	Animals with testes and a pouch with mammary glands instead of scrotum	Female animals with a pouch and mammary glands

The marsupial Y has the ability to turn the undifferentiated gonads into testes, but other aspects of sex differentiation seem to rely on the number of X chromosomes present. This is how we come across the above combinations in sex chromosome aneuploidies in marsupials.

### From monotremes to eutherian mammals

Intriguing as the marsupial X and Y chromosomes may be, it appears that they also exhibit close similarities to those encountered in man, as well as practically every other mammalian species. This observation has lead to the hypothesis of a common origin for the gonosomes of all current mammals. In an attempt to verify this theory, recent research related to sex determination in man has shifted its focus on the application of comparative genetics for different sex-specific sequences, both codal and non-codal, aiming to unravel the mystery of X and Y evolution [46,47]. It is important to note that the concept of a common ancestry for sex chromosomes has been originally proposed by S.Ohno as early as the 1960s and it was based on comparative observations on mammalian reproductive biology and gonadal physiology [48]. In fact, one of the authors (R.A.) has also had the fortune to contribute to the latter research as a member of the College de France research group, under the direct supervision of the late Professor Alfred Jost [49].

**Figure 15:**
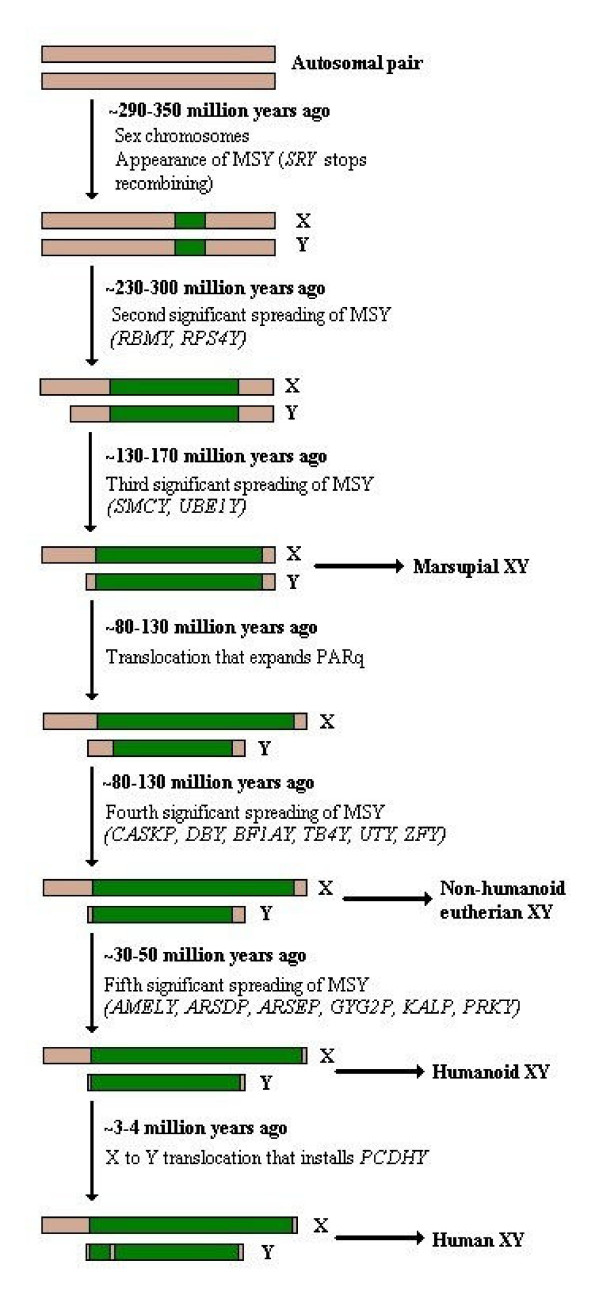
**The multistage model of sex chromosome evolution**. The mammalian X and Y chromosomes are thought to derive from a common initial autosomal pair. By a gradual process of genetic instability, which may have been related to failure in the recombination process, the chromosomes have begun to differ from each other. The first area to acquire a sex-specific role is considered to be the locus around the major sex determinant gene, i.e. SRY. Thus, in evolutionary lower mammals with a more conserved chromosomal content, such as monotremes, X and Y retain homology in all their length but for the SRY region. Subsequent stages of X-Y recombination failure have led to other, transient forms of X-Y structure, such as those observed in marsupials and primates. The greatest level of heterogeny is considered to be that found in modern humans.

According to a number of recent publications made by the research team of Dr D. Page and his colleagues (Jegalian and Page 1998, Jegalian and Lahn 2001) [50] the comparison of the nucleotide sequence of the X and Y chromosomes and their extend of homology in different mammals has lead to the conclusion that the development of discrete X and Y gonosomes is the result of, at least four, independent and recurrent, major stages of genomic evolution. In every stage, a failure in recombination between the homologous ancestors of the modern X and Y has been transferred to the next generations, leading to the continuous destabilization of their structure, permanent deletions in one of the chromosomes (the future Y) and a steady differentiation of their content, up to the current state, where homology remains only in the two remote pseudoautosomal areas in the telomeres of human gonosomes (see Figure 15).

The evolutionary model proposed by Page, Jegalian and Lahn in 1999 [51] suggests that more than 400 million years ago (i.e. before the evolution of the mammalian common ancestral line, roughly 300 million years ago), the common ancestor of modern reptiles and mammals retained an intact pair of autosomal, homologous genes instead of the X and Y. At the time, the two chromosomes where identical, sharing the same content and participating in autosomal recombination during the first meiotic division.

In the following evolutionary stage, it seems that failure in recombination during meiosis resulted in the reversal of part of one of the chromosomes (the future Y). This process inhibited further recombination attempts in the inverted region, since the presence of homologous sequences in opposite positions of the two chromosomes is a prerequisite for recombination. In future generations, this mistake wasn't repaired, thus instituting a permanent non-recombining area in the genome. As further failures in recombination followed, with a constant nucleotide loss in the unstable chromosome (Y), homology between the two chromosomes gradually decreased, until finally it was limited in the distant tips of the X and Y [52].

The first region where recombination failure must have occurred was the region of the *SRY *gene, which has since initiated a new role for genes of the Y chromosome, i.e. sex determination and sexual trait differentiation. This shift in gene function was the result of a long process of sequence variation, both in the encoding area and its regulatory elements. This evolutionary stage is placed 240–320 million years ago, an era consistent with the appearance of the ancestor of monotreme mammals (300 million years ago), animals known indeed to carry the *SRY *gene and only a limited area around it, where recombination between X and Y is not possible, contrary to the rest of their sequences [53].

A second stage in the formation of the X and Y is also attributed to a recombination failure, about 130–170 million years ago. At this time, the united mammalian line was separated in two others, namely the marsupial and placental lines.

As a result of the first two waves of X and Y differentiation discussed so far, the ability to estimate and regulate the level of gene expression in mammals was significantly hindered, since the processes of failed recombination, genomic reversal and deletion had already resulted in a significant loss of the sequences of the initial Y chromosome, contrary to the relatively intact X. In an attempt for proper dosage compensation between the two sexes, mammals developed the process of X chromosome inactivation (XCI) [54]. In effect, this process evolved in the course of time and in parallel with the continuous alteration in X and Y morphology and structure [55,56]. It is also possible that, initially, simpler mechanisms of dosage compensation where applied, such as the hyper-expression of the genes in the single X chromosome of males, as is the case in the modern fruitfly, *Drosophila melanogaster*.

A classic example of this gradual evolution of the dosage compensation strategies and, particularly, X inactivation, refers to the origin of LINE1 (long interspersed nuclear elements 1) sequences. These sequences interact with *XIST *(X inactivation specific transcript) RNA and, possibly other transitory proteins, forming a three-dimensional pattern that promotes gene silencing in the spreading stage of XCI. In placental mammals, LINE1 sequences have multiplied and spread throughout the Y chromosome, about 100-60 million years ago. This estimation suggests that the LINE1 sequences, an element necessary for stable XCI have evolved a long time after the separation of marsupials and placental mammals. Indeed, experimental data suggests that only the latter are capable of stable XCI, while the first possess an imprinted transient mechanism, lacking maintenance processes (e.g. methylation) [57].

Finally, a third stage in X-Y evolution is placed 80 to 130 million years ago and a final one 30 to 50 million years ago, coinciding with primate initial appearance. As in previous stages, failure in recombination is once again considered the promoter for these steps in the formation of the current X and Y.

A number of researchers focusing in comparative genomic studies and, especially, point mutations, attempt to clarify the exact evolutionary pattern for the gonosomes of every mammalian species. The addition of experimental data improves the estimation of the exact separation time for the ancestors of the major types of species on the earth, allowing the recognition of further sub-stages in the main pattern that has already been described. For instance, in the case of the X and Y, the initial recombination failure stage has been challenged, with some researchers proposing its substitution by two distinct phases, 350-290 and 290-230 million years ago [58]. In this case, it would be possible to assume a common origin for the gonosomes of mammals, reptiles and birds, preceding all other steps in the X-Y genomic evolution. However, no such procedure has been proven to this day. On the other hand, the classic concept of a common history for all mammal gonosomes and a completely discrete pattern for Z/W evolution in birds remains the most widely accepted in current evolutionary genetics.

The analysis of chromosome Y nucleotide sequence was an especially difficult task for the research teams involved in the Human Genome Project. According to a recent report (Skaletsky et al 2003), about 95% of the Y chromosome is now defined as the male specific region of the Y chromosome or MSY, for short. This area coincides with the previously described as non-recombining region of the Y chromosome or NRY. This change in terminology is not only aiming to emphasize the importance of the region for male sex determination, as it includes the *SRY *gene and its regulatory and downstream acting agents, but also attempting to correct a chronic misunderstanding, since this area is in fact participating in recombination. Interestingly, the latter doesn't involve the X, since no homology is present, but different parts of the MSY, in the form of a unique Y-Y internal recombination [59]. On the other hand, X-Y recombination is limited to the two pseudoautosomal regions, i.e. PAR1 and PAR2, thus leaving no part of the Y without the ability to participate in some form of recombination, as the term NRY would obviously suggest.

A further study of the sequences in the MSY allows a classification in three categories, each including areas of distinct structure, function and origin:

1. **X-degenerate genes. **This category includes genes deriving from the various stages of X-Y gradual differentiation proposed by Page and described so far. The term degenerate is used to emphasize their origin from the former ancestral autosome, which was equivalent in size to the X, before it gradually degenerated. One of the genes in this group is the *SRY *gene. In total, the category includes single copies of 14 pseudogenes and 13 genes, all having a homologue allele in the X. Most of these genes aren't expressed exclusively in a single, specific tissue. Their products are proteins produced in a variety of cells of the body, mediating non-sexual functions.

2. **X-transposed genes **[60]. These loci include a minimal number of genes and a large proportion of LINE1 sequences and other examples of non-coding DNA. Their homology with regions of the X chromosome leads to the conclusion that they must be a result of a distinct evolutionary process, significantly more recent than the stages proposed by Page. It is possible that these regions were directly translocated from X to Y, a process involving the parallel transfer of several intact genes.

3. **Ampliconic genes. **These genes exist in multiple copies on the Y, resulting from the replication of an initial copy. Apart from multiple copies per gene, this category also includes eight large palindromes. These genomic areas are characterized by inverted repeat sequences in their edges, while their centre appears to protect "hidden" genes and repeat, non-coding sequences [61]. In attempt to explain the creation of the palindromes the following pattern has been proposed:

1) An initial failure in recombination leads to the transfer of genes from autosomes to the ancestor of the modern Y

2) A series of amplification circles resulted in the presence of multiple copies for each one

3) The reversal of some of the copies has promoted the creation of the palindromes, trapping parts of the Y in between.

It is interesting to note that the formation of the palindromes increased the inherent stability of the Y, raising the question of its possible settlement in its current form, after thousands of years of degeneration and decay [62]. If this is indeed so, the whole theory of continuous Y deterioration as a cause of an increase in male infertility, due to the constant removal of genes essential for effective spermatogenesis, is seriously challenged [63].

**Figure 16:**
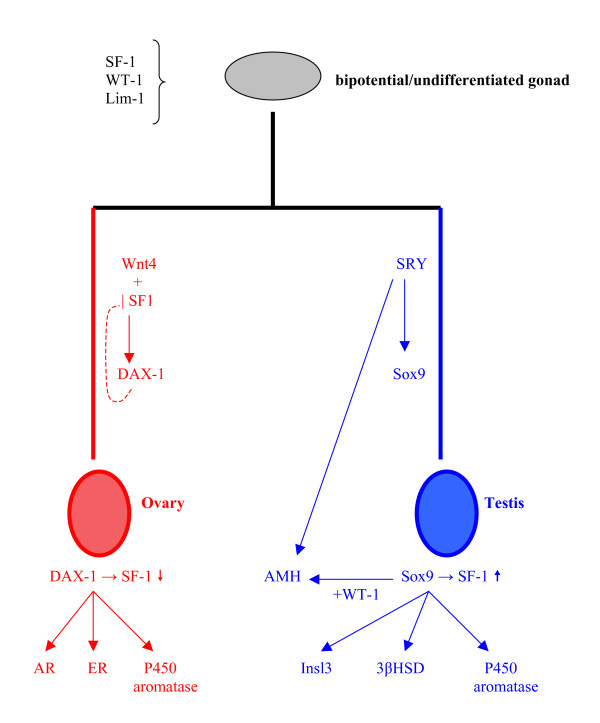
**Genetic model of sex determination in humans**. The formation of the undifferentiated/bipotential gonad is controlled by several genes acting simultaneously, such as *WT1*, *SF1 *and *Lim1*. Primary sex determination is based on the presence of the Y chromosome and its main sex-determining gene, *SRY*. In this case, *SOX9*, *FtzF1/SF1 *and *AMH *expression divert the gonad and the reproductive tract towards the male phenotype. This differentiation process is regulated by several other genes, including *DAX1*, *GATA4*, *FOXL2 *and, possibly, *DMRT1 and 2 *(not shown in the figure). In females, *SRY *absence allows gonadal development towards a female phenotype, mediated by genes such as *DAX1*, *Wnt4 *and *SF1*, resulting in aromatase upregulation. The exact role of *stra8 *(not shown) in this process remains to be clarified.

Sex determination in mammals has been more extensively studied than in any other species, most probably due to its direct relevance to human physiology and pathophysiology. A large number of genes have already been described and many more are expected to be added in the process, since the relevant research constantly reveals new players in the complex network of reactions related to sex determination (see Figure 16). Even in the common, bipotential gonad, the expression of several genes is considered crucial for subsequent development and normal sexual dimorphism. These include, among others, *WT1 *(Wilm's tumor 1), *FtzF1/SF1 *(Fushi tarazu factor 1/steroidogenic factor 1) and *Lim1*. Absence of any of these products at this stage, especially *WT1*, is inconsistent with further gonadal development and may also cause other malformations, e.g. affecting the adrenal gland and renal buds. Genes of the *wnt *family, such as *wnt4*, may also participate in the regulation of epithelial organization and epithelial-mesenchymal interactions in the area of the gonadal primordium [64].

*SRY *expression is the major sex-determining signal, since it is prerequisite for normal testis formation. Its role mimicks that of a molecular switch, since its peak expression is limited in a specific time period that is still considered sufficient to induce male-type differentiation of the reproductive system, via downstream gene action. The latter refers to several genes, including sox family members, *SF1 *(sex steroid regulation) and transcriptional factors, such as *GATA4*. *Sox *family genes share a common HMG box, similar to that observed in *SRY*, which is considered necessary for their action at a molecular level. The fact that members of the group have been detected in various species of vertebrates, such as fish (sox9) and all mammals (e.g. sox2 and sox 14 in monotremes) further emphasizes their importance for genetic sex determination [65]. The observation of this gene family's evolutionary conservation adds further credit to the multistage model of sex chromosome evolution described above, since *sox3 *has been proposed as the autosomal ancestor of *SRY*, which places it among the chronologically first sex-related genes in the common evolutionary history of all vertebrates [66].

In the female embryo, the Y chromosome is not present and, therefore, *SRY *is not expressed. The genetic cascade regulating female reproductive system differentiation is not as extensively studied as in men, but *DAX1 *(and its regulatory system, including genes such as *Wnt4 *and *SF1*) is generally considered as a significant player in this process, which is how it came to acquire the rather simplistic description of the "antitestis gene". Sex steroid production regulation is also important for the establishment of a normal female phenotype and it is mediated via SF1 expression and aromatase enzyme complex induction [64].

Two relatively recently described genes with a potential role in sex determination and differentiation are *DMRT1 *and *Stra8 *(stimulated by retinoic acid gene 8). The first has been already discussed in previous units as a conserved sex-related gene, bearing a DM domain originally studied in nematodes [67]. In humans, XY sex reversal in cases of 9p chromosome deletions have been attributed to impaired action of *DMRT1 *or its homologue, *DMRT2*. Still, their exact involvement in the sex determination circuit has not been clarified [68]. S*tra8*, on the other hand, is exclusively expressed in female germ cells and its presence signals their sexual gradual differentiation, in an anterior to posterior direction. However, it has not yet been established whether the gene's product directly induces sex determination towards the female pathway, or rather acts a simple marker of this phenomenon, without active participation in the process per se [69,70].

### Hormonal and epigenetic regulation of sex determination

Hormonal regulation of sex determination is a vast research field in modern reproductive endocrinology. In fact, recent advances have resulted in a more generalized study of sexual dimorphism, with the discovery that differences expand to far more than the reproductive organs, including visceral tissues and the brain. The study of sex steroid concentrations and the presence of their receptors in various parts of the CNS has already been attempted in various species, including mammals and reptiles. After all, the role of androgens and estrogens in sexual differentiation in vertebrates is a classic concept that modern research data has only supported and expanded, rather than criticize [5,28]. For instance, aromatase regulation appears to be the final target in the sex determination circuit of several turtles. This has been proven by the experimental work of C.Pieau and colleagues, using aromatase inhibitors to effectively block feminization of the embryos [26].

Other scientists have even attempted to suggest sex steroids as a driving force in X-Y evolution. According to JM Howard (2002), androgens may be a major regulator of X-Y differentiation [4]. Although increased testosterone may be beneficial for fertility, constant exposure to high quantities may result in spermatogenic arrest. The *DAZ *gene of the Y is believed to have appeared 30–40 million years ago as a means to maintain spermatogenesis. [71] In females, increased testosterone levels caused evolutionary pressure and limited the total population, as only few of them survived and transferred their DNA in next generations, a process detected by mitochondrial DNA comparative studies. This is an example of the bottleneck phenomenon, and due to its reference to females, it has been described as the mitochondrial Eve hypothesis [4,71,72]. A number of studies in comparative genomic support this theory, such as the results of the research team lead by Hammer (1995) [73]. Increased testosterone levels acting in descendants of these women has resulted in a second wave of evolutionary pressure, surpassed by the maintenance of spermatogenesis by a duplication of the DAZ gene, about 50.000–200.000 years ago [74]. These stages of evolutionary pressure and limitation of the total population may explain the large-scale homology of the regions of the Y chromosome among all modern males (Adam phenomenon) [75]. Failure to provide sufficient evidence, such as the description of all androgen gene targets, their exact importance for male fertility and the degree of their conservation among modern men has not allowed to adequately verify the validity of this theory to this date.

Moreover, sex determination may be related to other, non-hormonal phenomena as well. For instance, immunological parameters and paracrine messages/cytokines may be involved in aromatase regulation, as some relevant initial data indicate [29,30]. In addition, sex has been proposed to be associated with selective cell proliferation. This view is supported by U. Mittwoch and is largely based on the comparative observation of male and female gonadal development in different successive stages and for a number of different model organisms [1]. If this is indeed so, it could be the result of sex steroid regulation, thus sharing some common ground with the abovementioned theories. Alternatively, there could be a completely independent pathway of mitotic induction, implicating a number of growth factors. The description of several sex-related genes conserved in various species may support this view, since sex steroids alone may not be sufficient to explain these genes' action, especially in the case of invertebrates. On the other hand, epigenetic regulation of the sexual phenotype has been proposed, which means that the products of these genes (or their downstream aftermath) could influence DNA replication and/or transcription by direct contact within the nucleus. This mechanism may be evaluated by the analytical description of all epigenetic changes occuring at a chromatin level during the various stages of normal sex differentiation and their comparison with observations made in individuals with sex distortions [5].

## Conclusion

Sex determination is a crucial process in developmental biology. Its accurate regulation is a prerequisite for reproductive success and, therefore, the continued survival of a species. Since reproduction is also the function that determines the categorization of specific populations in the same or different species, the analysis of the specific molecular patterns that this process may follow is crucial for the comprehension of the detailed biochemical background mediating and maintaining the phenotypical variety observed at a macroscopical level. This is also useful for the explanation of the mechanism of infertility, since in many cases the disorder is caused by a genetic default.

Contemplating the above mechanisms as a whole, it is clear that they exhibit many differences (e.g. environmental contribution, number of genes involved, known primary sex-determinant or simultaneous action of different genes), but intriguing similarities as well. Among the latter one may briefly point at: a) the central role of aromatase regulation for female vertebrates. This similarity could also be generalized to include all sex steroids and their regulators, such as *SF1*. One should not fail to detect the homology of *SF1 *encoding gene, *FtzF1 *to Drosophila's *fushi tarazu*, which has not yet been adequately explained in terms of either evolutionary origin or gene function.

b) the action of *sox *family proteins in all mammals and some other vertebrates, such as fish. This category includes the primary sex determinant of all mammals, namely *SRY*, which might justify its proposed evolutionary history from an original autosome homologue, namely *sox3*.

c) the conservation of genes bearing a DM or LIM domain. This is a relatively new finding, but the fact that these products spread from dipterans and nematodes to humans must imply some degree of coherence in their regulatory mechanisms.

Whether to establish inter-relating patterns of evolution or simply for the sake of the knowledge that can be gleaned from understanding these vastly diffident mechanisms, these differences and similarities will definitely continue to hold the interest of the scientific community for years to come.

## Abbreviations

**sl-CSD: **single-locus complementary sex determination

***csd*: **complementary sex determiner

***sxl*: **sex-lethal

***sis-a,b,c*: **sisterless-a,b,c

***dpn*: **deadpan

***da*: **daughterless

***emc*: **extramacrochaetae

***her*: **hermaphrodite

***snf*: **sans fille

***DSX*: **doublesex

***SEX-1*: **signal element on X 1

***FOX-1*: **feminizing on X 1

***XOL-1*: **XO lethal 1

***TRA-1*: **transformer 1

***HER-1*: **hermaphrodite 1

**TSP: **thermosensitive period

**Cyt P450: **cytochrome P450

***DAX1*: **dosage-sensitive sex reversal 1

***DMRT1*: **doublesex- and mab-3-related transcription factor 1

***SOX9*: **SRY related HMG box 9

***AMH*: **Anti-Müllerian Hormone

***SRY*: **sex determining region of the Y chromosome

***DMY*: **DM domain of the Y chromosome

***FIGa*: **factor in the germ line a

***FTzF1*: **fushi tarazu factor 1

***SF1*: **steroidogenic factor 1

***FET1*: **female-expressed transcript 1

***ASW*: **avian sex-specific on W chromosome

***WPKCI*: **W chromosome protein kinase C inhibitor

***ZPKCI*: **Z chromosome protein kinase C inhibitor

**XCI: **X-chromosome inactivation

**LINE1: **long interspersed nuclear elements

***XIST*: **X inactivation specific transcript

**MSY: **male specific region of the Y chromosome

**NRY: **non- recombining region of the Y chromosome

**PAR-1, 2: **pseudo autosomal region 1, 2

***WT1*: **Wilm's tumor 1

***Stra8*: **stimulated by retinoic acid gene 8

***DAZ*: **deleted in azoospermia

## Competing interests

To the authors' best knowledge, no competing interests of any nature arise from the current publication

## Authors' contributions

The three authors have participated equally in all the steps of the preparation of the submitted manuscript. In particular, the bibliographical research, the compilation of the first draft, the proofreading and editing processes were undertaken by the contributing authors in a series of relevant meetings in the Department of Histology and Embryology, Medical School, Athens University. In addition, all the images of the manuscript were prepared using image analysis software by P. Manolakou and later edited by the other two authors.
